# Guidelines for Designing Social Robots as Second Language Tutors

**DOI:** 10.1007/s12369-018-0467-6

**Published:** 2018-01-25

**Authors:** Tony Belpaeme, Paul Vogt, Rianne van den Berghe, Kirsten Bergmann, Tilbe Göksun, Mirjam de Haas, Junko Kanero, James Kennedy, Aylin C. Küntay, Ora Oudgenoeg-Paz, Fotios Papadopoulos, Thorsten Schodde, Josje Verhagen, Christopher D. Wallbridge, Bram Willemsen, Jan de Wit, Vasfiye Geçkin, Laura Hoffmann, Stefan Kopp, Emiel Krahmer, Ezgi Mamus, Jean-Marc Montanier, Cansu Oranç, Amit Kumar Pandey

**Affiliations:** 1 Centre for Robotics and Neural Systems, Plymouth University, Plymouth, UK; 2 IDLab – imec, Ghent University, Ghent, Belgium; 3 Tilburg Center for Cognition and Communication, Tilburg University, Tilburg, The Netherlands; 4 Cluster of Excellence Cognitive Interaction Technology, Bielefeld University, Bielefeld, Germany; 5 Department of Special Education: Cognitive and Motor Disabilities, Utrecht University, Utrecht, The Netherlands; 6 Department of Psychology, College of Social Sciences and Humanities, Koç University, Istanbul, Turkey; 7 SoftBank Robotics, Paris, France

**Keywords:** Social robot, Second language learning, Robot tutor, Human–robot interaction

## Abstract

In recent years, it has been suggested that social robots have potential as tutors and educators for both children and adults. While robots have been shown to be effective in teaching knowledge and skill-based topics, we wish to explore how social robots can be used to tutor a second language to young children. As language learning relies on situated, grounded and social learning, in which interaction and repeated practice are central, social robots hold promise as educational tools for supporting second language learning. This paper surveys the developmental psychology of second language learning and suggests an agenda to study how core concepts of second language learning can be taught by a social robot. It suggests guidelines for designing robot tutors based on observations of second language learning in human–human scenarios, various technical aspects and early studies regarding the effectiveness of social robots as second language tutors.

## Introduction

One of the goals of Human–Robot Interaction (HRI) is to research and develop autonomous social robots as tutors that are able to support children learning new skills effectively through repeated interactions. To achieve this, the interactions between child and robot should be pleasant, challenging, and pedagogically sound. Interactions need to be pleasant for children to enjoy, challenging so that children remain motivated to learn new skills, and pedagogically sound to ensure that children receive input that optimises their learning gain. One domain in which robots for learning are developed is second language (L2) tutoring (e.g., [1, 33, 64]). While much progress has been made in this field, there has not been an effective one-on-one L2 tutoring programme that can be structurally applied in educational settings for various language communities.

The L2TOR project^1^ (pronounced as ‘el tutor’) aims to bridge this gap by developing a lesson series that helps preschool children, around the age of 5 years, learn basic vocabulary in an L2 using an autonomous social robot as tutor [8]. In particular, we develop one-on-one, personalised interactions between children and the SoftBank NAO robot for teaching English to native speakers of Dutch, German, and Turkish, and for teaching Dutch or German to Turkish-speaking children living in the Netherlands or Germany. To ensure a pedagogically sound programme, lessons are being developed in close collaboration with developmental psychologists and pedagogists.

Personalising the interactions between child and robot is crucial for successful tutoring [45]. Personalisation can be achieved by creating some initial common ground between child and robot, and by having the robot adapt to the individual progress of children. Constructing initial common ground helps to promote long-term interactions between child and robot [33], and can be achieved by framing the robot as a peer and by explaining (dis)similarities between robots and humans. However, to keep children motivated to learn, it is important to keep the learning targets within the child’s *Zone of Proximal Development* [70]. Throughout the lessons the target should be sufficiently challenging for the child: not too challenging as this may frustrate the learner and not too easy as this may bore the learner. Moreover, interactions should be designed such that the robot provides a scaffold that allows the child to acquire the desired language skills. For instance, by providing non-verbal cues (e.g., gestures) that help to identify a word’s referent or by providing appropriate feedback, it is possible for children to reinforce successfully acquired skills or to correct suboptimal (or wrong) skills.

The L2TOR approach relies on the current state-of-the-art in HRI technology, which offers promising opportunities, but also poses major challenges. For instance, NAO has the ability to produce speech in various languages, making it possible for the robot to address the child in both the native language (L1) and in the L2. However, at present, automatic speech recognition (ASR) for child speech is not performing to a sufficiently reliable standard, and thus using ASR is currently infeasible [37]. This not only limits the ability to rely on verbal interactions since the robot is unable to respond to children’s speech, but it also limits the ability to monitor and respond to children’s L2 productions. Hence, our design has to find ways to work around such technological limitations.

The paper aims to present a number of guidelines that help researchers and developers to design their own social robot, especially for, though not necessarily limited to, L2 tutoring. After a brief review of L2 learning from a developmental psychology point of view, Sect. 3 reviews some previous research on language tutoring using social robots. In Sect. 4, we will present our guidelines relating to pedagogical considerations, child–robot interactions and interaction management. These issues will be discussed in light of some of our early experiments. Section 5 discusses our approach to evaluating the L2TOR system, which is designed to demonstrate the (potential) added value of using social robots for L2 tutoring.

## Second Language Learning

Learning an L2 is important in today’s society. In the European Union (EU), for example, 54 percent of the population can hold a conversation in at least two languages, and 25 percent are able to speak three languages [20]. Consequently, L2 teaching has become an essential part of primary education. In 2002, the EU proposed a multilingualism policy of teaching an L2 to all young children. The policy suggests every European citizen learns practical skills in at least two languages aside from their L1 [4]. According to a recent survey, the vast majority of European citizens (98 percent of the respondents in this survey) believe that mastering a foreign language is useful for the future of their children [20].

Preschool years are vital for L2 learning, because later academic success depends on early language skills [29]. For children learning English as their school language, their English vocabulary size predicts their performance in English reading tests [57]. Although learning an L2 comes naturally for some children, for many others it is a challenge that they must overcome. For children from immigrant families or minority communities, the language used at school is often different from the language used at home. These children, thus, not only start learning the school language later than their peers, but also continue to receive relatively less input in each of their languages [30]. Hence, novel ways to expose children to targeted L2 input must be considered.

Patterns of L2 learning largely mirror those of L1 learning, which requires both the quantity and the quality of language input to be sufficient [27]. Children do not learn language just by listening to speech; rather, interactive experience is essential [39]. L2 learning is no exception, and several factors such as interactivity must be considered (see [38] for a review). In addition to quantity, socio-pragmatic forms of interaction involving joint attention, non-verbal interaction, feedback, and temporal and semantic contingencies are expected to contribute to L2 learning [3, 9, 59, 66]. However, there are also some notable differences between L1 and L2 learning. For example, in L2 education it is important to consider from whom children are learning the L2. Place and Hoff [56] found that hearing English from different speakers and the amount of English input provided by native speakers is critical for learning English as L2. Another notable difference between L1 and L2 learning is that children may rely on their L1 when learning an L2 (e.g., [75]). Thus, we may need to be cautious about factors such as negative transfer or interference, in which some concepts and grammar in the L2 are hard to acquire because children are thinking in their L1 [67].

When children are learning more than one language, the amount of input a child hears in each language predicts vocabulary size in each language [30, 55]. Bilingual children tend to have a smaller vocabulary size in each language compared to their monolingual peers [54], although the combined or conceptual vocabulary size of both languages is often equal to that of monolinguals [31, 54]. The amount of language input also affects language processing speed and trajectories of vocabulary learning, and thus early language input may have cascading effects on later language learning. Hurtado et al. [32] found that the amount of language input bilingual children receive at 18 months of age predicts their speed of recognizing words and the size of their vocabulary at 24 months. To properly foster development of two or more languages, adults must carefully consider a good balance between languages [67].

Although both monolingual and bilingual children monitor and respond to social pragmatic cues, bilingual children have heightened sensitivity to those non-linguistic cues, probably due to an early communicative challenge they face because of less than perfect mastery in one of the languages [74]. Brojde et al. [10] found that bilingual children rely more on eye gaze than their monolingual counterparts when learning novel words. Yow and Markman [76] also demonstrated that 3- and 4-year-old bilingual children were better at understanding and using gestures and gaze direction to infer referential intent. Thus, especially for children with advanced L2 knowledge, we may be able to boost their learning process by making use of these pragmatic cues.

As the demand for early L2 education increases, the usage of additional teaching opportunities in terms of educational tablet games, or electronic vocabulary trainers becomes more and more important to increase the quantity of L2 input. Moreover, especially with regard to young children, the consideration of embodied technologies (e.g., virtual agents or robots) seems reasonable, because they invite intuitive interactions that would add to the quality of the L2 input. The question then becomes: how should such a robot be designed?

## Robots for Language Tutoring

In recent years, various projects have started to investigate how robot tutors can contribute to (second) language learning. In this section, we review some of these studies, focusing on: (a) the evidence that robots can promote learning; (b) the role of embodiment in robot tutoring; and (c) the role of social interactions in tutoring.

### Learning from Robots

There has been an increased focus on how social robots may help engage children in learning activities. Robots have been shown to help increase interaction levels in larger classrooms, correlating with an improvement in children’s language learning ability [22]. How best to apply this knowledge in the teaching of a foreign language has been explored by different researchers from various perspectives. Alemi et al. [1] employed a social robot as an assistant to a teacher over a 5-week period to teach English vocabulary to Iranian students. They found that the class with the robot assistant learned significantly more than that with just the human teacher. In addition, the robot-assisted group showed improved retention of the acquired vocabulary. This builds on earlier findings by [33] where a 2-week study with a robot situated in the classroom revealed a positive relation between interacting with a robot and vocabulary acquisition. Further results by [64] also confirm that the presence of a robot leads to a significant increase in acquired vocabulary. Movellan et al. [50] selected 10 words to be taught by a robot, which was left in the children’s classroom for 12 days. At the end of the study, children showed a significant increase in the number of acquired words when taught by the robot. Lee et al. [42] further demonstrated that robot tutoring can lead not just to vocabulary gains, but also improved speaking ability. In their study, children would start with a lesson delivered by a computer, then proceed to pronunciation training with a robot. The robot would detect words with an expanded lexicon based on commonly confused phonemes and correct the child’s pronunciation. Additionally, the children’s confidence in learning English was improved.

All of these studies show the capacity of various robots as tutors for children (with the children’s age ranging from 3 to 12 years old) learning an L1 or L2 ‘in the wild’. However, what exactly is it that gives robots the capacity for tutoring? Moreover, how does this compare to other digital technologies, such as tablets and on-screen agents? Is it merely the embodiment of the robot, or rather the quality of social interactions? These questions are explored in the following sections.

### Embodiment

The impact of embodiment and social behaviour for children learning English as their L1 has been explored in a laboratory setting. Neither [24] nor [71] found significant differences due to the embodiment of the robot in their studies on children’s vocabulary acquisition. However, this may be due in part to methodological limitations. Gordon et al. [24] only found an average of one word learned per interaction, leaving very little room for observing differences; similarly [71] only compared the learning of six words. These studies were conducted with children between the ages of 3 and 8 years. The relatively small gains are therefore quite surprising, due to the speed at which children at this age acquire language [40]. Given the non-significant results or the small effect sizes in these studies, it is difficult to draw conclusions on what could make robot language tutoring effective.

Rosenthal-von der Pütten et al. [58] found that language alignment, i.e., the use of similar verbal patterns between interacting parties, when using an L2 appears to not be affected when using a virtual robot as opposed to a real one. Participants completed a pre-test and were then invited for a second session at a later date. During the second session the participants were asked to play a guessing game with an agent, either the real NAO robot or a virtual representation of one. The study reported whether the participants used the same words as the agent, but no significant difference was found. This may be due to some issues with the experimental design: the authors suggest the post-test was given straight after a relatively long session with the agent, and participants may have been fatigued.

Moriguchi et al. [49] looked at age differences for young children and how they learned from a robot compared to a person. Children between the ages of 4 and 5 years were taught using an instructional video: one group of children was shown a video in which a human taught them new words, while another group of children was shown a video with the same material, but using a robot tutor. While children aged 5 were able to perform almost as well when taught by a robot, those aged 4 did not seem to learn from the robot at all. It is unknown as to whether this result would transfer to the use of a physically-present robot, rather than one shown on a video screen.

These studies above do not provide support that the mere physical presence of the robot has an advantage for language learning. However, there is evidence for the physical presence of a robot having a positive impact on various interaction outcomes, including learning [46]. The lack of a clear effect of a physical robot on language learning might be due to a scarcity of experimental data. However, it is also likely that the effectiveness of robot tutors lies not in their physical presence, but instead in the social behaviour that a robot can exhibit and the motivational benefits this carries. This is explored in the next section.

### Social Behaviour

Social behaviour has previously been studied in the context of children learning languages. Saerbeck et al. [60] explored the impact of ‘socially supportive’ behaviours on child learning of the Toki Pona language, using an iCat robot as a tutor. These behaviours included verbal and non-verbal manipulations which aimed to influence feedback provision, attention guiding, empathy, and communicativeness. It was found that the tutor with these socially supportive behaviours significantly increased the child’s learning potential when compared to a neutral tutor. This study used a variety of measures including vocabulary acquisition, as other studies have, but also included pronunciation and grammar tests. Another study which did not only consider vocabulary acquisition was [26]. French and Latin verb conjugations were taught by a NAO robot to children aged 10 to 12 years old. In one condition, the robot would look towards the student whilst they completed worksheets, but in the other, the robot would look away. Although gaze towards the child was predicted to lead to greater social facilitation effects, and therefore higher performance, this was not observed.

Kennedy et al. [36] investigated the effects of verbal immediacy on the effect of learning in children. A NAO was used to teach French to English-speaking children in a task involving the gender of nouns and the use of articles ‘le’ and ‘la’. A high verbal immediacy condition was designed in which the robot would exhibit several verbal immediacy behaviours, for example calling the child by name, providing positive feedback, and asking children how they felt about their learning. When contrasted with a robot without this behaviour, no significant learning differences were observed. However, children showed significant improvement in both conditions when comparing pre- and post-test scores, and were able to retain their acquired knowledge as measured by means of a retention test. This suggests that the particularities of robot behaviour do not manifest themselves in the short-term, but could be potentially be observed over the longer term.

In [2], a robot acted as a teaching assistant for the purpose of teaching English to Iranian students. A survey found that students who were taught by the robot were significantly less anxious about their lessons than those that were not. This was thought to be due to a number of factors, including the fact that the robot was programmed to make intentional mistakes which the students could correct, which could have made students less concerned about their own mistakes.

### Summary

In summary, promising results have been found for the use of robots as constrained language tutors for children and adults, with the presence of the robot improving learning outcomes [1, 2, 33, 64]. However, the impact of robot embodiment in this context has not been explored in depth, leaving an important question largely unanswered: do robots hold an advantage over tablets or virtual characters for language tutoring? The impact of social behaviour is also less clear, with some positive results [60], but also inconclusive results [26]. Robots open up new possibilities in teaching that were previously unavailable, such as the robot taking the role of a peer. By having an agent that is less like a teacher and more like a peer, anxiety when learning a new language could be reduced [2]. Despite an increasing interest, there are still relatively few studies that have considered robot language tutoring, leaving space to explore novel aspects of language learning.

## Designing Robot Tutoring Interactions for Children

Several design issues with respect to robot-guided L2 tutoring have to be considered before an evaluation of robot-child tutoring success is possible. In particular, multiple design choices have to be considered to create pleasant, challenging, and pedagogically sound interactions between robot and child [69]. First, we will discuss pedagogical issues that ensure optimal conditions for language learning. Second, we will present various design issues specifically relating to the child–robot interactions. Finally, we will discuss how to manage personalised interactions during tutoring. The section builds on some related work as well as various studies conducted in the context of the L2TOR project.

### Pedagogical Issues

It is imperative to understand how previous research findings can be put into practice to support successful L2 acquisition. Although the process of language learning does not drastically differ between L1 and L2, there are a few notable differences as we already discussed in Sect. 2. For the L2TOR project a series of pedagogical guidelines was formulated, based on existing literature and pilot data collected within our project. These guidelines concern: (a) age differences; (b) target word selection; (c) the use of a meaningful context and interactions to actively involve the child; and (d) the dosage of the intervention. These specific aspects were chosen based on a review of the literature showing that they are the most crucial factors to consider in designing an intervention for language teaching in general and specifically L2 (see e.g., [29, 51]).

#### Age Effects

From what age onward can we use social robots to support L2 learning effectively? From a pedagogical point of view, it is desirable to start L2 tutoring as early as possible, especially for children whose school language is an L2, because this could bridge the gap in language proficiency that they often have when entering primary school [29]. Various studies have targeted children as young as 3 years focusing on interactive storytelling in the L1 [22] or on L2 tutoring [73]. However, preschool-aged children (3 to 5 years old) undergo major cognitive, emotional and social developments, such as the expansion of their social competence [15]. So, whereas older children may have little difficulty engaging in an interaction with a robot, younger children may be more reliant on their caregivers or show less engagement in the interaction. Therefore, we may expect that child–robot interactions at those ages will also present some age-related variation. Clarifying these potential age differences is essential as, in order to be efficient, interactive scenarios with robots must be tailored to the diverging needs of children.

In [6], we sought to determine whether there are age-related differences in first-time interactions with a peer-tutor robot of children who have just turned 3 and children who are almost 4 years old. To this end, we analysed the engagement of 17 younger children ($M_{age}=3.1$ years, $SD_{age}=2$ months) and 15 older children ($M_{age}=3.8$ years, $SD_{age}=1$ month) with a NAO robot as part of the larger feedback experiment discussed in Sect. 4.2.6. These children first took part in a group introduction to familiarise them with the NAO robot; a week later they had a one-on-one tutoring session with the robot. We analysed the introductory part of this one-on-one session, which consisted of greeting, bonding with, and counting blocks with the robot. All speech was delivered in Dutch, except for the target words (i.e., ‘one’, ‘two’, ‘three’, and ‘four’), which were provided in English. We analysed the children’s engagement with the robot as measured through eye-gaze towards the task environment (robot and blocks) compared to their gazes outside the task environment (experimenter, self, and elsewhere), as this is suggested to indicate how well the child is “connected” with the task [62].

In short, the analyses revealed that the older children gazed significantly longer towards the robot than the younger children, and that the younger children spent more time looking elsewhere than the older children. Moreover, the average time the older children maintained each gaze towards the robot was longer than that of the younger children.

It is possible that the 3-year-olds have trouble being engaged with a language learning task, but it may also be that the NAO robot is somewhat intimidating for 3-year-olds. As such, for them either group interactions [22] or a more “huggable” robot (e.g., Tega) [73] could be more appropriate. Moreover, [49] also found children at the age of 5 years to be more responsive to robot tutoring. Drawing from these findings about 3-year-olds, combined with experiences from other pilots with 4- and 5-year-olds, we decided to develop the L2TOR tutoring system for 5-year-olds, as they generally appear to feel more comfortable engaging one-on-one with the robot than 3- and 4-year-olds.

#### Target Words

Another important aspect to consider is what words are taught. Previous research recommends that vocabulary items should be taught in semantic clusters and embedded in a conceptual domain [11, 51]. For L2TOR, three domains were chosen: (a) number domain: language about basic number and pre-mathematical concepts; (b) space domain: language about basic spatial relations; and (c) mental states domain: language about mental representations such as ‘being happy’ and propositional attitudes such as ‘believe’ or ‘like’. These domains were selected for their feasibility, as well as their relevance and applicability in L2 tutoring sessions in a preschool setting. Appropriate words to be taught for each domain are words that children should be familiar with in their L1, as the goal of the intervention is not to teach children new mathematical, spatial, and mental state concepts, but rather L2 labels for familiar concepts in these three domains. This will enable children to use their L1 conceptual knowledge to support the learning of L2 words. To select appropriate target words and expressions that children are familiar with in their L1, a number of frequently used curricula, standard tests, and language corpora were used. These sources were used both for identifying potential targets, and for checking them against age norms to see whether they were suitable for the current age group (for more details, see [53]). Thus, target words selection should be based both on semantic coherence and relevance to the content domain and on children’s L1 vocabulary knowledge.

#### Meaningful Interaction

An additional aspect of L2 teaching is the way in which new words are introduced, which may come to affect both learning gains as well as the level of engagement. Research has indicated that explicit instruction on target words in meaningful dialogues involving defining and embedding words in a meaningful context yields higher word learning rates than implicit instruction through fast mapping (i.e., mapping of a word label on its referent after only one exposure) or extracting meaning from multiple uses of a word in context as the basic word learning mechanisms [48, 51]. Therefore, for the L2TOR project, an overall theme for the lessons was selected that would be familiar and appealing to most children, and, as such, increase childrens engagement during the tutoring sessions. This overall theme is a virtual town that the child and the robot explore together, and that contains various shops, buildings, and areas, which will be discovered one-by-one as the lesson series progresses. All locations are familiar to young children, such as a zoo and a bakery. During the lessons, the robot and the child discover the locations, and learn L2 words by playing games and performing simple tasks (e.g., counting objects or matching a picture and a specific target word). The child and the robot are awarded a star after each completed session, to keep children engaged in the tasks and in interacting with the robot. Thus, the design chosen for L2TOR is thought to facilitate higher learning gains as it involves explicit teaching of target words in a dialogue taking place in a meaningful context. Moreover, this design should facilitate engagement as it involves settings that are known and liked by children.

#### Dosage of Language Input

The final pedagogical aspect that was identified in the literature concerns the length and intensity, or dosage, of the intervention. Previous research has shown that vocabulary interventions covering a period of 10 to 15 weeks with one to four short 15- to 20-min sessions per week are most effective. As for the number of novel words presented per session, the common practice is to offer 5 to 10 words per session, at least in L1 vocabulary interventions [47]. However, not much is known about possible differences between L1 and L2 interventions with regard to this aspect. Therefore, to determine the number of target words to be presented in the L2TOR project lesson series, a pilot study was conducted. In this study, we taught English words to one hundred 4- and 5-year-old Dutch children with no prior knowledge of English. We started by teaching the children 10 words; when these were established, more words were added. The results showed that, for children to learn any of these words at all, the maximum number of L2 words that could be presented in one session was six. We also found that a high number of repeated presentations of each word was necessary for word learning: each word in our study was presented 10 times. Yet, children’s accuracy rates in the translation and comprehension tasks in our study were lower than in earlier work on L1 learning. A possible explanation might be that the items included in the study were relatively complex L2 words (e.g., adjectives like ‘empty’) rather than concrete nouns such as ‘dog’ or ‘house’. These items are probably more difficult for children who had no prior exposure to the target language. However, within the L2TOR project the choice was made to include these relatively complex items given their relevance for L2 learning within an academic context [52]. Thus, it was decided that in all the lessons included within the L2TOR project a maximum of six words will be presented in each lesson and each word will be repeated at least ten times throughout the lesson.

### Child–Robot Interaction Issues

Not only pedagogical issues need to be considered when designing a social robot tutor, but also other issues relating to how the interactions between the robot and child should be designed. As mentioned, we focus on how to design the interactions to be pleasant, challenging, and pedagogically sound. In this section, we discuss six aspects that we deem important: (a) first encounters; (b) the role of the robot; (c) the context in which the interactions take place; (d) the non-verbal behaviours and (e) verbal behaviours of the robot; and (f) the feedback provided by the robot.

Before elaborating on these guidelines, it is important to remind the reader that in L2TOR, we are designing the robot to operate fully autonomously. Ideally, this would include the possibility to address the robot in spoken language and that the robot can respond appropriately to this. However, as previously mentioned, current state-of-the-art in speech recognition for child speech does not work reliably. Kennedy et al. [37] compared several contemporary ASR technologies and have found that none of them achieve a recognition accuracy that would allow for a reliable interaction between children and robots. We have therefore decided to mediate the interactions using a tablet that can both display the learning context (e.g., target objects) and monitor children’s responses to questions. This has the consequence that the robot cannot monitor children’s L2 production autonomously, but it can monitor children’s L2 comprehension through their performance with respect to the lesson content presented on the tablet.

#### Introducing the Robot

The first encounter between robot and child plays a large role in building the child’s trust and rapport with the robot, and to create a safe environment [72], which are necessary to facilitate long-term interactions effectively. For example, [21] has shown that a group introduction in the kindergarten prior to one-on-one interactions with the robot influenced the subsequent interactions positively. Moreover, [72] have shown that introducing the robot in a one-to-many setting was more appreciated than in a one-on-one setting, because the familiarity with their peers can reduce possible anxiety in children.

We, therefore, developed a short session in which the robot is introduced to children in small interactive groups. In this session, the experimenter (or teacher) first tells a short story about the robot using a picture book, explaining certain similarities and dissimilarities between the robot and humans in order to establish some initial common ground [14, 33]. During this story, the robot is brought into the room while in an animated mode (i.e., turned on and actively looking around) to familiarise the children with the robot’s physical behaviour. The children and the robot then jointly engage in a meet-and-greet session, shaking hands and dancing together. We observed in various trials that almost all children were happy to engage with the robot during the group session, including those who were a bit anxious at first, meaning these children likely benefited from their peers’ confidence. Although we did not test this experimentally, our introduction seems to have a beneficial effect on children’s one-on-one interaction with the robot.

#### Framing the Robot

One of the questions that arises when designing a robot tutor is: How should the robot be framed to children, such that interactions are perceived to be fun, while at the same time be effective to achieve language learning? We believe it is beneficial to frame the robot as a peer [5, 7, 24], because children are attracted to various attributes of a robot [33] and tend to treat a robot as a peer in long-term interactions [64]. Moreover, framing the robot as a peer could make it more acceptable when the flow of the interaction is suboptimal due to technical limitations of the robot (e.g., the robot being slow to respond or having difficulty interpreting children’s behaviours). In addition, framing the robot as a peer who learns the new language together with the child sets the stage for learning by teaching [64].

While the robot is framed as a peer and behaves like a friend of the child, the tutoring interactions will be designed based on adult-like strategies to provide the high quality input children need to acquire an L2 [39], such as providing timely and sensible non-verbal cues or feedback. So, in L2TOR we frame the robot as a peer, it behaves like a peer, but it scaffolds the learning using adult-like teaching strategies.

#### Interaction Context

To facilitate language learning, it is important to create a contextual setting that provides references to the target words to be learned. The embodied cognition approach, on which we base our project, states that language is grounded in real-life sensorimotor interactions [28], and consequently predicts that childrens interactions with real-life objects will benefit vocabulary learning [23]. From this approach, one would expect children to learn new words better if they manipulate physical objects rather than virtual objects on a tablet, as the former allows children to experience sensorimotor interactions with the objects. However, for technical reasons discussed earlier, it would be convenient to use a tablet computer to display the context and allow children to interact with the objects displayed there. The question is whether this would negatively affect learning. Here, we summarise the results from an experiment comparing the effect of real objects versus virtual objects on a tablet screen on L2 word learning [68]. The main research question is whether there is a difference in L2 vocabulary learning gain between children who manipulate physical objects and children who manipulate 3D models of the same objects on a tablet screen.

In this experiment, 46 Dutch preschoolers ($M_{age}=5.1$ years, $SD_{age}=6.8$ months; 26 girls) were presented with a story in Dutch containing six L2 (English) target words (i.e., ‘heavy’, ‘light’, ‘full’, ‘empty’, ‘in front of,’ and ‘behind’). These targets were chosen as children should benefit from sensorimotor interactions with objects when learning them. For example, learning the word ‘heavy’ could be easier when actually holding a heavy object rather than seeing a 3D model of this object on a tablet screen. Using a between-subjects design, children were randomly assigned to either the tablet or physical objects condition. During training, the target words were each presented ten times by a human. Various tests were administered to measure the children’s knowledge of the target words, both immediately after the training and one week later to measure children’s retention of the target words.

**Fig. 1:**
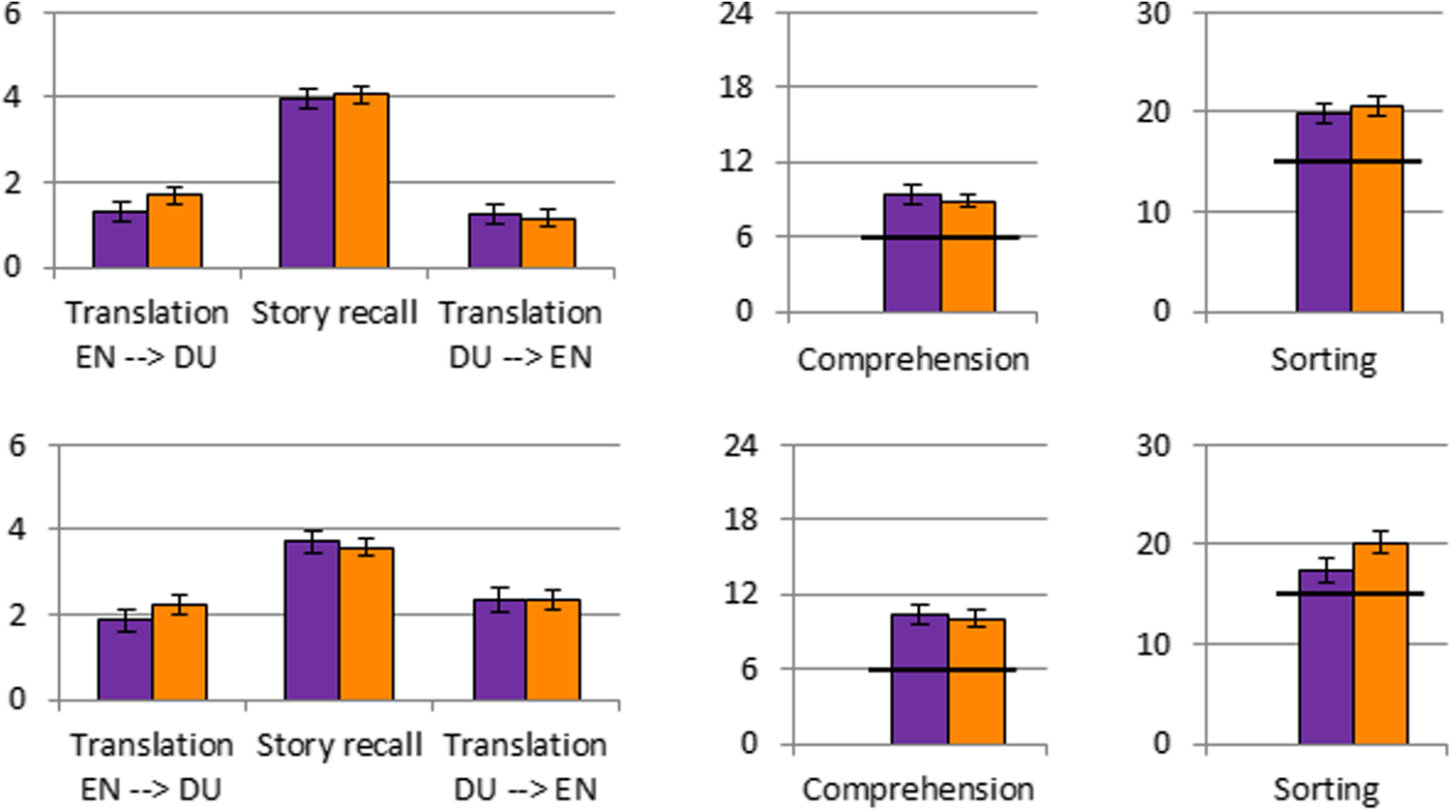
Mean accuracy scores on the direct post-test (top) and the delayed post-test (bottom). Purple bars refer to the object condition; orange bars to the tablet condition. Reprinted from [68]. (Color figure online)

**Fig. 2:**
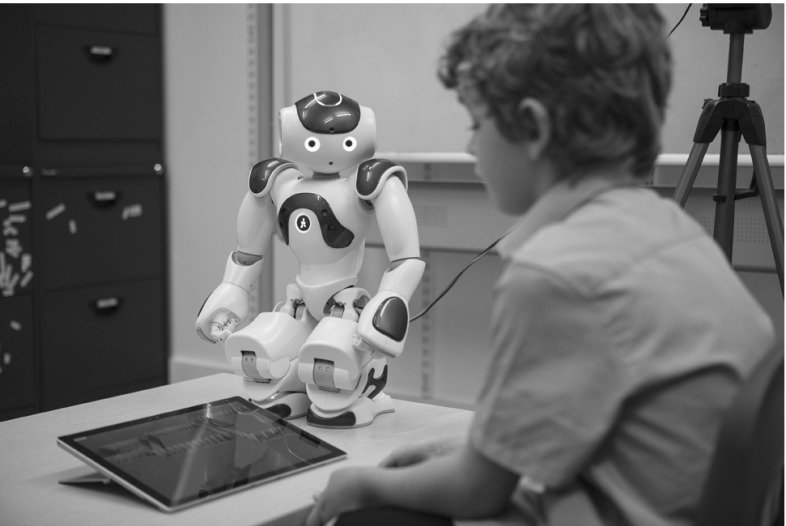
The L2TOR setup includes the NAO robot standing to the side of the child with a tablet in between them

Independent-samples t-tests revealed no significant differences between using a tablet or physical objects on any of the tasks, as indicated by childrens mean accuracy scores on the direct and delayed post-tests (see Fig. 1; all *p* values $> .243$). In the receptive tests (the comprehension task and sorting task), children scored significantly above chance level (indicated by the black line), irrespective of condition (all *p* values $<.001$). Interestingly, in both conditions, the mean scores on the Dutch-to-English translation task were higher for the delayed post-test than for the immediate post-test (both *p* values $<.001$), possibly indicating some sort of “sleep effect”. These findings indicate that it does not matter much whether the context is presented through physical objects or a tablet computer.

Displaying the context (i.e., target objects) on a tablet does not seem to hamper learning, which is convenient, since using a tablet makes designing contexts more flexible and reduces the need to rely on complex object recognition and tracking. Because of this, the lessons in the L2TOR project are displayed on a tablet, which is placed between the child and the robot (see Fig. 2). This tablet not only displays the target objects (e.g., a set of elephants in a zoo), but also allows children to perform actions on these objects (e.g., placing a given number of elephants in their cage). Since at present ASR for children is not performing reliably [37], the robot cannot monitor children’s pronunciation or other verbal responses. We therefore focus on language comprehension rather than language production and use the tablet to monitor comprehension. The use of a tablet in the interaction allows us to monitor the child’s understanding of language and to control the interaction between child and robot.

#### Non-verbal Behaviour

Human language production is typically accompanied by non-verbal cues, such as gestures or facial expressions. It is therefore not surprising that research in children’s language development has shown that the use of gestures facilitates L2 learning in various ways (e.g., [25, 59, 65]). Gestures could take the form of deictic gestures, such as pointing to refer to physical objects near the child, or of iconic gestures used to emphasize physical features of objects or actions in a more representational manner. Such iconic gestures help to build congruent links between target words and perceptual or motor information, so learners may benefit not only from observing gestures, but also by way of execution, such as enactment and imitation [23, 25].

Due to its physical presence in the child’s referential world, a robot tutor has the ability to use its physical embodiment to its advantage when interacting with the child, for example, through the manipulation of objects in the real world, or simply through the use of gestures for various communicative purposes. We believe that the robot’s ability to use gestures is one of the primary advantages of a robot as tutor compared to a tablet computer, since it can enrich the language learning environment of the child considerably by exploiting the embodiment and situatedness of the robot to facilitate the child’s grounding of the second language.

Even though a growing body of evidence suggests that non-verbal cues, such as gestures aid learning, translating human’s non-verbal behaviour to a robot like NAO remains a challenge, mostly due to hardware constraints. For instance, the NAO robot is limited by its degrees of freedom and constraints with respect to its physical reach, making it unable to perform certain gestures. Motions may sometimes seem rigid, causing the robot’s movements to appear artificial rather than human-like. Especially when certain subtleties are required when performing a gesture, such shortcomings are not desirable. A noteworthy complication comes with the NAO’s hand, which has only three fingers that cannot move independently of one another. This makes an act such as finger-counting, which is often used for the purpose of explaining numbers or quantities, practically impossible.

This, thus, requires a careful design and testing of appropriate referential gestures, because otherwise they may harm learning [35].

#### Verbal Behaviour

One potential advantage of using digital technologies, such as robots, is that they can be programmed to speak multiple languages without an accent. However, NAO’s text-to-speech engines do generate synthetic voices and have few prosodic capacities. Yet, studies have shown that children rely on prosodic cues to comprehend spoken language (e.g., [16]). Moreover, adults typically use prosodic cues to highlight important parts of their speech when addressing children. In addition, the lack of facial cues of the NAO robot may potentially hinder the auditory-visual perception processes of both hearing-impaired and normal-hearing children [19]. These limitations pose the question to what extent children can learn the pronunciation of L2 words sufficiently well.

To explore this, a Wizard-of-Oz (WoZ) experimental pilot was devised using the NAO robot and a tablet for tutoring and evaluating English children counting up to five in German. The task involved multiple steps to gradually teach children to count, in L2, animals shown on screen. First, the robot-tablet concept was introduced, with the robot describing content displayed on the tablet screen, and the children were trained on how and when to provide answers by means of touching images on said screen. The children then proceeded with the main task, which involved the counting of animals, first in English and later in German. The interaction was managed by using multiple utterances from a WoZ control panel in order to prompt the children to give the answer only after they were asked to. The WoZ operator triggered appropriate help and feedback from the robot to the child when required. Finally, at the end of the task, the robot asked the children to count up to five again with the robots help and then without any help at all. The purpose of this step was to evaluate whether the children were able to remember the pronunciation of the German numbers and if they were able to recall them with no support.

**Fig. 3:**
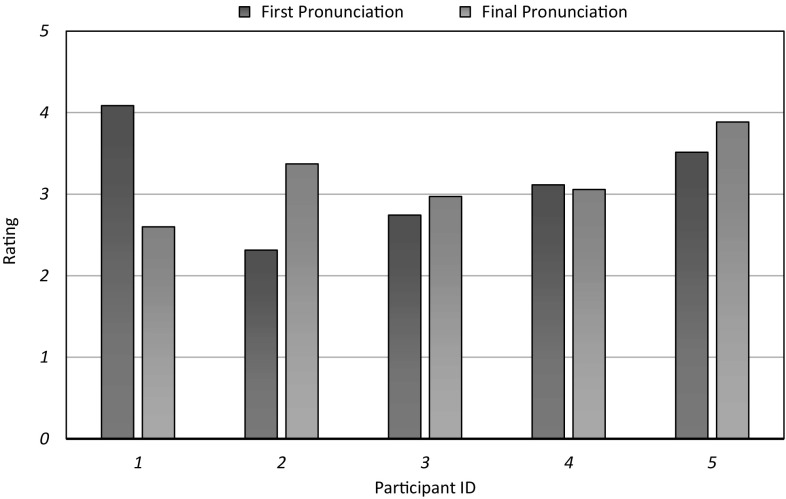
Pronunciation ratings from seven German native speakers for 5 child participants. Three of the children improve over the course of the interaction, although one child has initially accurate pronunciation that drops over time, possibly due to fatigue

Voice and video recordings were used to record the interactions with five children aged 4 to 5 years old. The first and final repetitions of the children pronouncing the German words were recorded and rated for accuracy on a 5-point Likert scale by seven German-native coders; intraclass correlation $ICC(2,7)=.914$, indicating “excellent” agreement [13]. Based on these ratings, our preliminary findings are that repetitions generally improve pronunciation. Several children initially find it hard to pronounce German numbers but they perform better by the end (Fig. 3). This may be because some children had trouble recalling the German numbers without help. We believe that the task needs updating to improve the children’s recall (by, for example, including more repetitions). In addition, it should be noted that children generally find it difficult to switch from English to German.

To conclude, children can learn the pronunciation of the L2 from the robot’s synthetic voice, but we should compare this to performance ratings of children that have learned the L2 from native speakers. It is worth noting that they seem to have some reservation speaking a foreign language, but whether or not this is due to the presence of the robot is unknown.

#### Feedback

A typical adult-like strategy known to support language learning is the use of appropriate feedback [3]. Adult caregivers tend to provide positive feedback explicitly (e.g., ‘well done!’) and negative feedback implicitly by recasting the correct information (e.g., ‘that is the rabbit, now try again to touch the monkey’). However, evidence suggests that a peer does not generally provide positive feedback and that they provide negative feedback explicitly without any correction (e.g., ‘no, that is wrong!’). So, when the robot is framed as a peer, should it also provide feedback like a peer?

To explore this, we carried out an experiment to investigate the effect the type of feedback has on children’s engagement [17, 18]. In the experiment, sixty-five 3-year-old children (30 boys, 35 girls; $M_{age}=3.6$ years, ${\textit{SD}}_{age}=3.6$ months) from different preschools in the Netherlands participated. Six children stopped with the experiment before it was finished and were excluded from the data. The children were randomly assigned to one of three conditions, varying the type of feedback: adult-like feedback, peer-like feedback, and no feedback. The adult-like feedback of the robot used reformulations to correct the children in case they made a mistake (e.g., ‘three means *three*’, where the text in italics represents what the robot said in the L2, here English; the rest was said in the L1, here Dutch) and positive feedback (‘well done!’) when children responded correctly. In the peer-like condition, only explicit negative feedback without correction was provided whenever children made a mistake (‘that is wrong!’) and no feedback was provided when they responded correctly. In the no feedback condition, the robot simply continued with the next task without providing any feedback.

During the experiment, the robot taught the native Dutch-speaking children counting words one to four in English. The interaction consisted of an introductory phase followed by the tutoring phase. During the introductory phase, the target words (i.e., ‘one’, ‘two’, ‘three’, and ‘four’) were described and associated with their concept in sentences such as ‘I have *one* head’, ‘I have *two* hands’, ‘I have *three* fingers’, and ‘there are *four* blocks’. We analysed the introductory phase as part of the age-effects study reported in Sect. 4.1.1. In the tutoring phase, the robot asked the child to pick up a certain number of blocks that had been placed in front of them. All instructions were provided in Dutch and only the target words were provided in English. After the child collected the blocks, the robot provided either adult-like feedback, peer-like feedback, or no feedback depending on the experimental condition assigned to the child.

As a result of the relatively low number of repetitions of the target words over the course of the interaction, we did not expect to find any effects with respect to learning gain. However, the objective was not to investigate the effect feedback has on learning, but rather on the child’s engagement with the robot as an indicator of learning potential [12]. As for the age-effect study, we analysed engagement by annotating the children’s eye-gaze towards the robot, human experimenter, to the blocks, and elsewhere, and measured the average time children maintained their gaze each time they looked at one of these targets.

**Fig. 4:**
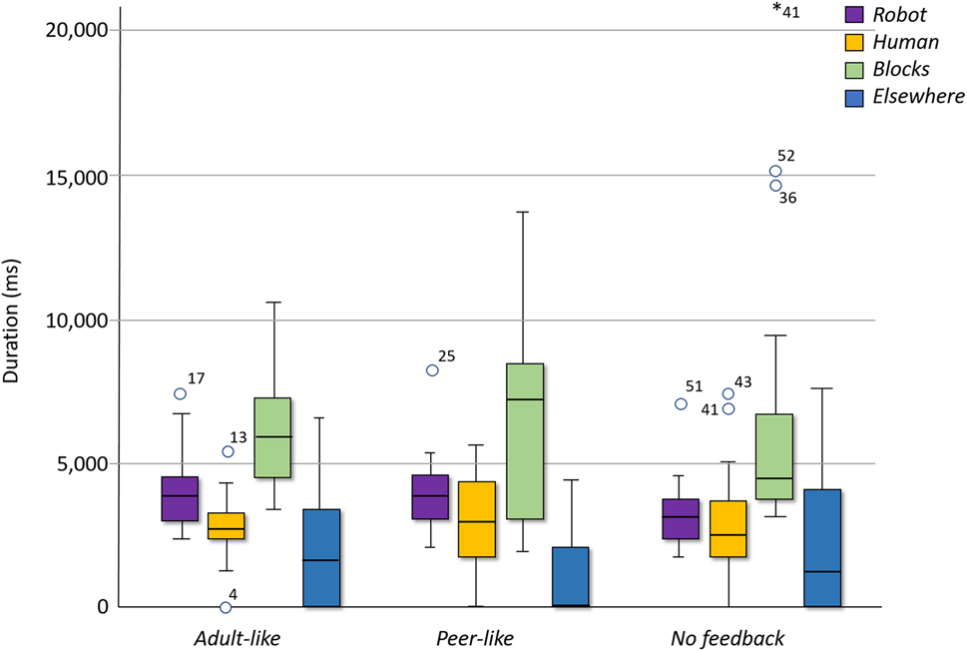
Mean duration per gaze to the robot, blocks, experimenter, and elsewhere for the three feedback conditions

Results from a repeated measures ANOVA indicated that, on average, the children maintained their gaze significantly longer at the blocks and the robot than at the experimenter, regardless of their assigned condition (see Fig. 4).

However, we did not see any significant differences in the gaze duration across the three conditions. As such, the way the robot provides feedback does not seem to affect the engagement of the child with the robot. This would suggest that, as far as the child’s engagement with the robot and task is concerned, it does not matter how the robot provides feedback or whether the robot provides feedback at all. Hence, the choice for the type of feedback that the robot should give can, thus, solely be based on the effect feedback has on learning gain. Future work will investigate which type of feedback is most effective for learning.

### Interaction Management

#### Objective

To realise robot-child tutoring interactions that provide a pleasant and challenging environment for the child, while at the same time being effective for L2 learning, interaction management plays a crucial role.

As children typically lose interest when a lesson is either too easy or too difficult, personalisation of the lessons to each child’s performance is very important. The tutor has to structure the interaction, needs to choose the skills to be trained, must adjust the difficulty of the learning tasks appropriately, and has to adapt its verbal and non-verbal behaviour to the situation. The importance of personalised adjustments in the robot’s behaviour has been evidenced in research showing that participants who received personalised lessons from a robot outperformed others who received non-personalised training [5, 45]. Suboptimal robot behaviour (e.g., too much, too distracting, mismatching, or in other ways inappropriate) can even hamper learning [35]. Therefore lessons should be adapted to the knowledge state (i.e., level) of the child [70].

Along these lines, the L2TOR approach is to personalise language tutoring in HRI by integrating knowledge-tracing into interaction management [61]. This adaptive tutoring approach is realised in a model of how tutors form mental states of the learners by keeping track of their knowledge state and selecting the next tutoring actions based on their likely effects on the learner. For that purpose, an extended model based on Bayesian Knowledge Tracing was built that combines knowledge tracing (what the learner learned) and tutoring actions in one probabilistic model. This allows for the selection of skills and actions based on notions of optimality: the desired learner’s knowledge state as well as optimal task difficulty.

#### Proposed Model

A heuristic is employed that maximises the beliefs of all skills while balancing the single skill-beliefs with one another. This strategy is comparable to the vocabulary learning technique of *spaced repetition* as implemented, for instance, in the Leitner system [43]. For the choice of actions, the model enables simulation of the impact each action has on a particular skill. To keep the model simple, the action space only consists of three different task difficulties (i.e., easy, medium, hard).

#### Results

As an evaluation, the model was implemented and tested with a robot language tutor during a game-like vocabulary tutoring interaction with adults ($N=40$) [61].

We adopted the game ‘I spy with my little eye’. In this game, the NAO robot describes an object which is displayed on a tablet along with some distractors, by referring to its descriptive features in an artificial L2 (i.e., “Vimmi”). The student then has to guess which object the robot refers to. The overall interaction structure, consisting of five phases (i.e., opening, game setup, test-run, game, closing), as well as the robot’s feedback strategies were based on our observations of language learning in kindergartens. After the tutoring interaction, a post-test of the learned words was conducted.

**Fig. 5:**
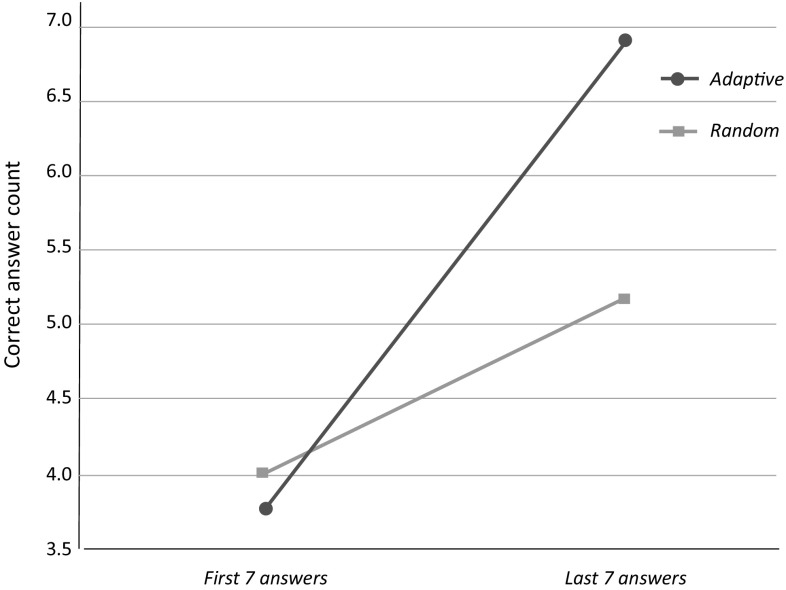
Mean numbers of correct answers at the beginning (first 7) and end (last 7) of the interaction in the different conditions. Adapted from [61]

The results revealed that learners’ performance improved significantly during training with the personalised robot tutor (Fig. 5). A mixed-design ANOVA with training phase as a within-subjects factor and training type as between-subject factor demonstrated a significant main effect of training phase ($F(1,38)=66.85, p<.001, \eta ^2=.64$), such that learners’ performance was significantly better in the final phase as compared to the initial phase. Crucially, participants who learned in the adaptive condition had a higher number of correct answers as compared to the control condition ($F(1,38)=6.52, p=.02, \eta ^2=.15$). Finally, the interaction between training phase and type was also significant ($F(1,38)=14.46, p=.001, \eta ^2=.28$), indicating that the benefit of the adaptive training developed over time.

**Table 1:** Results of both post-tests (L1-to-L2 and L2-to-L1): Means (M) and standard deviation (SD) of correct answers grouped by the experimental conditions

	Adaptive (A)		Control (C)	
	M	SD	M	SD
L1-to-L2	3.95	2.56	3.35	1.98
L2-to-L1	7.05	2.56	6.85	2.48

Adapted from [61]

**Fig. 6:**
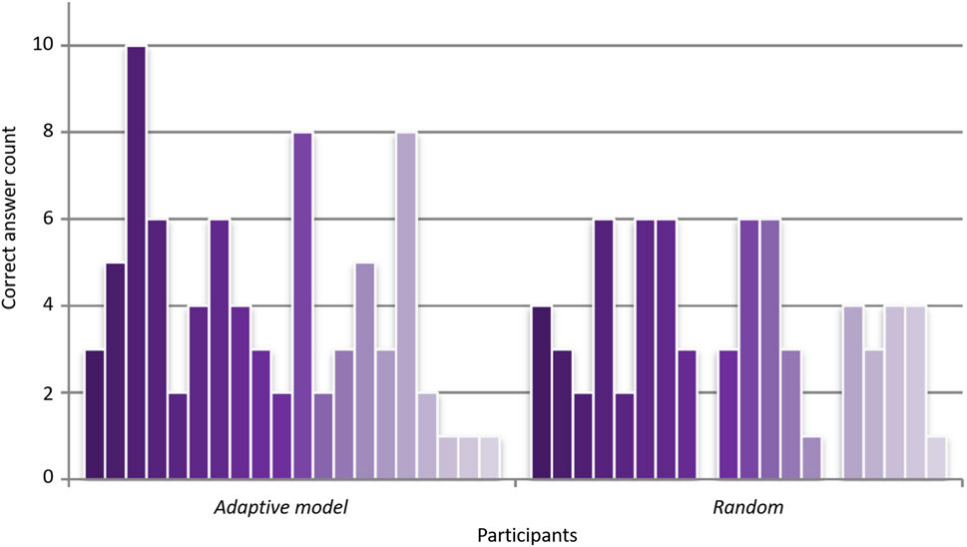
Participant-wise amount of correct answers grouped by the different conditions for the L1-to-L2 post-test. Adapted from [61]

The results of the post-test did not show significant differences between the two conditions, which may be due to the way in which responses were prompted during the training sessions and post-test (Table 1). In the training sessions participants saw pictures relating to the meaning of the to-be-learned words, whereas in the post-test they received a linguistic cue in form of a word they had to translate. Although no main effect of training type emerged in the post-test, some details are nevertheless worth mentioning. In the L1-to-L2 post-test, a maximum of ten correct responses was achieved by participants of the adaptive-model condition, whereas the maximum in the control condition were six correct answers. Moreover, there were two participants in the control condition who did not manage to perform any L1-to-L2 translation correctly, while in the adaptive-model condition, all participants achieved at least one correct response (see Fig. 6).

#### Outlook

This basic adaptive model will be extended by further integrating skill interdependencies as well as affective user states. Both have already been shown to improve learning [34, 63]. In addition, the model can, and is meant to, provide a basis for exploiting the full potential of an embodied tutoring agent, and will therefore be advanced to the extent that the robot’s verbal and non-verbal behaviour will adapt to the learner’s state of knowledge and progress. Specifically, it aims to enable dynamic adaptation of (a) embodied behaviour such as iconic gesture use, which is known to support vocabulary acquisition as a function of individual differences across children (cf. [59]); (b) the robot’s synthetic voice to enhance comprehensibility and prosodic focusing of content when needed; and (c) the robot’s socio-emotional behaviour depending on the learners’ current level of motivation and engagement.

## Evaluation Framework for Robot L2 Tutoring

In this section, we discuss our plans for evaluating our robot-assisted L2 vocabulary intervention. While this section describes future plans rather than already completed work, it also provides guidelines for evaluating tutoring systems similar to the L2TOR system. The first step in an evaluation is the development of pre- and post-tests designed to assess children’s learning of the targeted vocabulary through comprehension and translation tasks, as well as tasks assessing deep vocabulary knowledge (i.e., conceptual knowledge associated with a word). Not directly targeted but semantically-related vocabulary will also be assessed, as well as general vocabulary and other skills related to word learning (e.g., phonological memory). This is important as children learn not only the words directly used, but can also use these words to bootstrap their further vocabulary learning in the same as well as related domains [51].

In addition to assessing children’s L2 word learning, we will evaluate the word learning process during the interactive sessions between children and the robot by observing, transcribing, and coding video-taped interactions. Measures will include children’s and the robot’s participation and turn-taking, the type of questions, recasts and expansions, the semantic contingency of responses and expansions, and the coherence and length of episodes within the sessions. All these aspects are known to promote language learning [9, 44]. Therefore, it is important to evaluate how these processes are taking place within the context of language learning with a social robot.

Finally, given the importance of motivation, we will observe how children comply with the robot’s initiatives and instructions, how involved they are in the intervention, and to what extent they express positive emotions and well-being during the lessons [41]. The intervention will consist of multiple sessions, such that children’s learning, motivation, and interaction with a social robot can be judged over time.

The design of the evaluation study will be based on a comparison between an experimental and a control group. The experimental group will be taught using the social robot while the control group will receive a placebo training (e.g., non-language activity with the robot). This design is very common in educational research as it enables testing whether children who participate in an educational programme (L2TOR in this case) learn more or just as much as children who follow the normal curriculum. Additionally, learning gains with the robot will be compared to learning gains using an intelligent tutoring system on a tablet, to test the additional value of a social robot above existing technology used in education. In evaluating the robot-supported program developed within L2TOR, our aim is not only to assess the effectiveness of the specific tutoring by the L2TOR robot, but also to provide recommendations for further technological development and guidelines for future use of social robots in (L2) language tutoring situations.

## Conclusion

In this paper, we have presented guidelines for designing social robots as L2 tutors for preschool children. The guidelines cover a range of issues concerning the pedagogy of L2 learning, child–robot interaction strategies, and the adaptive personalisation of tutoring. Additional guidelines for evaluating the effectiveness of L2 tutoring using robots were presented.

While the benefits of social robots in tutoring are clear, there are still a range of open issues on how robot tutors can be effectively deployed in educational settings. The specific focus of this research programme –tutoring L2 skills to young children– requires an understanding of how L2 learning happens in young children and how children can benefit from tutoring. Transferring the tutoring to social robots has highlighted many questions: should the robot simulate what human tutors do? Should the robot be a peer or a teacher? How should the robot blend L1 and L2? How should feedback be given?

Our aim is to develop an autonomous robot: this incurs several complex technical challenges, which cannot currently be met by state-of-the-art AI and social signal processing. ASR of child speech, for example, is currently insufficiently robust to allow spoken dialogue between the robot and the young learner. We propose a number of solutions, including the use of a tablet as an interaction-mediating device.

Our and our colleagues’ studies show that social robots hold significant promise as tutoring aids, but a complex picture emerges as children do not just learn by being exposed to a tutoring robot. Instead, introducing robots in language learning will require judicious design decisions on what the role of the robot is, how the child’s learning is scaffolded, and how the robot’s interaction can support this.

## Footnotes

1 http://www.l2tor.eu.
